# A comparative assessment of oral health-related quality of life of children born with orofacial clefts in Sudan and their caregivers'

**DOI:** 10.1186/s12903-021-01514-2

**Published:** 2021-03-23

**Authors:** Mecheala Abbas Ali, Shaza K. Abass, Elwalid Fadul Nasir

**Affiliations:** 1 Department of Orthodontics and Pedodontics, Faculty of Dentistry, University of Science and Technology, Khartoum, Sudan; 2 Department of Orthodontics and Pedodontics, Faculty of Dentistry, University of Khartoum, Khartoum, Sudan; 3 Preventive Dentistry Department, College of Dentistry, King Faisal University, Alahsa, Kingdom of Saudi Arabia

**Keywords:** Oral health-related quality of life, Cleft lip and/or palate, Child oral health impact profile, Caregivers', Children, Sudan

## Abstract

**Background:**

Cleft lip and palate(CL/P)is the most common orofacial malformation affecting one in every 700–1000 newborns worldwide. The aim of the study wasto evaluate the impact of CL/P on health- related quality of life (OHRQoL) in Sudanese children and the level of concordance between caregivers' and children and to investigate correlates of the caregivers' perceptions of OHRQoL with that of their children.

**Methods:**

The sample consisted of 75 children with clefts (age range 8–16 years), (46 male, 29 female) and their caregivers' attending University of Science and Technology Dental Teaching Hospital. The children and their caregivers' were interviewed separately. The interview consisted of 38 questions adopted from the COHIP (Arabic version).The level of concordance between caregivers' and children was compared using paired *t*-tests and intraclass correlations. Predictive validity was assessed using Pearson correlations and linear regression analyses.

**Results:**

The translated COHIP and its subscales, had Cronbach' alphas score ranged between (0.65 and 0.75) for caregivers' and children with cleft. COHIP scores for children and caregivers' were (89.41 ± 19.97) and (94.34 ± 19.52) respectively. Caregivers' and children differed significantly in the overall COHIP and oral symptoms subscale. There were high correlations between caregivers' and children ranged from (0.63 to 0.87). The correlation between all subscales was statistically significant (*p* = 0.05).

**Conclusions:**

Caregivers' had higher perceptions of oral symptoms and overall COHIP compared to their children using the Arabic version of the COHIP. Caregiver's reports have to be considered complementary to the reports of their children themselves.

## Background

The quality of life concept has been of increasing importance in healthcare among many disciplines [1]. The World Health Organization (WHO, 1998, p. 17) defines the quality of life as "individuals’ perception of their position in life in the context of the culture and value system where they live, and in relation to their goals, expectations, standards, and concerns. The impact of oral health on quality of life is usually referred to as oral health-related quality of life (OHRQoL) [2].

OHRQoL is recognized as a multidimensional concept, comprising both the presence and absence of oral disease and psychological aspects of oral health [3].Children with orofacial clefts (OFCs) were reported to having a lower quality of life scores compared to children with no facial defects [4]. In Sudan the incidence of CL/P is about 0.9/1000 child births [5]. Children with OFCs showed few differences in health-related quality of life scores, oral symptoms, and emotional well-beingcompared to children with dental caries [6]. It was acknowledged that poor oral health could have a substantial adverse effect on the quality of life, as people may, for example, have difficulty in eating, feeling ashamed of their teeth, or experience pain. Apparently, oral health is an essential part of general health and well-being [7].

Health professionals who work with children with cleft lip/palate (CL/P) report that these children have modest decreases in health-related quality of life (HRQoL) [8]. The treatment of these children with CL/P usually involves several complicated and extensive procedures from infancy into adulthood. Still, the eventual goal is to achieve a good functional and aesthetic result, which will allow for psychological and social well-being [9].

Children and adolescents provide consistent information regarding their own (OHRQoL) when appropriate questionnaire techniques are used. Even though few scales specifically designed to measure OHRQoL have been developed [10–12], the Child Oral Health Impact Profile (COHIP) was developed to assess OHRQoL in terms of oral health, functional well-being, social-emotional well-being, school environment, and self-image in children [13]. It has excellent reliability and validity [14]. Research has demonstrated the usefulness of obtaining caregivers' reports of the child's OHRQoL in addition to the report completed by the child [15].

The importance of facial appearance, time away from school, and symptomology may be over-or under-estimated by caregivers' [16]. Caregivers' are also likely to have biases and expectations, which can affect the quality of life (QoL) score of their children. When children have chronic or extreme health conditions, caregivers' tend to report more negative QoL than that reported by the child [17, 18]. Bos et al. [19] found that Parents’ reports on their children’s OHRQoL were not in agreement with reports of cleft lip and/or palate patients. In comparison, many previous studies reported no difference between children and caregivers' perceptions of their child's OHRQoL in any subscales and overall scores of COHIP [20–22]. Evaluation of OHRQoL response agreement between children and their caregivers' can also be helpful as healthcare providers seek methods of estimating the OHRQoL of children who are either mentally or physically unable to complete a questionnaire [22].

The burden of OFCs on individuals, families, and society can be alleviated through understanding the impact of OFCs on the well-being of affected ones. It is also essential to identify their healthcare needs and make changes in healthcare practices and public policies to improve the well-being of the victims [20].

The aim of the present study was to evaluate the impact of CL/P on the OHRQoL of Sudanese children using the Arabic version of the COHIP. Second, to assess the possible differences between the OHRQoL scores reported by children and caregivers'. Third, to investigate the correlates of the caregivers' perceptions of OHRQoL with that of their children.

## Methods

### Subjects

This is a cross-sectional study carried out among children with oral clefts attending the cleft clinic of the orthodontic department at the University of Science and Technology dental teaching hospital. Selection criteria were; the child with (CL/P) between 8 and 16 years of age, able to speak and communicate, non- syndromic, and not physically or mentally handicapped. The children satisfying the inclusion criteria were identified, and the study purposes were explained to the caregivers' and their children. Following parental consent and child assent, 75 children with oral clefts and their parents were included. We used the translated COHIP questionnaire, which consists of two parallel questionnaires, one for children and one for caregivers'. The Arabic language was used.

### Reliability and validity of the Arabic version of the COHIP

Two experts who are fluent in English and Arabic translated the English version of the questionnaire into the Arabic language. Later, another two who were also fluent in Arabic and English back translated it into English. Modifications to the questionnaire were made as needed to guarantee comprehension. Six university dental staff-members were asked to compare the English versions, and they agreed that there is no significant difference between the two versions. To test the reliability, fifteen participants were re–interviewed within two weeks of their initial interview. A reliability test was performed. The Intraclass correlation coefficient [ICC] was ˃ 0.8.

### Data collection

To maintain confidentiality, subjects satisfying the inclusion criteria were assigned a number. The principal investigator (M. A.A) interviewed the Children and their caregivers' separately. The interview consisted of 38 questions adopted from the COHIP (Arabic version). The items in "Oral symptoms" subscale measured specific oral symptoms that are not necessarily related to one another. "Functional Well-being" measured information about the child's capability to carry out specific everyday activities such as speaking clearly, chewing, and sleeping. "Social-Emotional Well-being" is concerned with anxiety, shyness, and peer interactions. "School-Environment Well-being" related to tasks associated with the school environment. "Self-image" addressed positive feelings about self. "Treatment Expectancy" measured expectations regarding the treatment process and outcomes. "Global Health" assessed overall feelings about oral and systemic health.

Each item was valued on a 5-point scale ranging from 0 to 4 ("never" = 0, "almost never" = 1, "sometimes" = 2, "fairly often" = 3, and "almost all the time" = 4) the 28 negatively-worded items were reversed. The positively scored items were the six items of self-image.

In addition to the COHIP, positively formulated questions were added for clinical use regarding global health perceptions and treatment expectations. The additional questions were on a 5-point scale, “totally disagree” = 0, “disagree” = 1 “don’t disagree/don’t agree” = 2, “agree” = 3, and “totally agree” = 4.High COHIP scores reflected a positive OHRQoL while lower scores reflected a negative OHRQoL. Subscales scores were calculated by summing the responses of the items specific to the subscale [13]. The overall OHRQoL score was computed by summing the subscales scores. Treatment expectation scores and global health responses were not included in the overall COHIP scale because these items are relevant only when the COHIP is used as part of a treatment assessment. The total score of the scale is usually between 0 and 136. One extra question, asking about the general heath of the child was added to both questionnaire. This item had similar response categories, ranging from 0 to 4.

### Statistical analysis

Statistical analysis was performed using Statistical Package for Social Sciences (SPSS) version 20. A significance level was set at α ≤ 0.05. The internal consistency of the questionnaire as a whole and each of the subscales was examined using Cronbach's Alpha.

Descriptive statistics (frequency, mean, median, standard deviation) for the collected variables were calculated. Responses between caregivers' and children were compared using a Paired-sample *t*-test. ICCs were computed to indicate a measure of agreement. Finally, the predictive value of the COHIP for general health was evaluated by using Pearson's correlation coefficients.

### Ethical approval

Caregivers' and children were informed about the research, and written informed consent from the caregivers' and the child assent were obtained. Ethical and Research Committee of Faculty of Dentistry, University of Science and Technology approved all study procedures. The committee’s reference number was No. UST/FD/ERC.21.11.2016.All methods were carried out in accordance with relevant guidelines and regulations.

## Results

Table 1 shows the sample profile of both children and caregivers'.

**Table 1:** General characteristic of the sample

Subjects (n = 70)	N (%)
Gender of children	
Male	46 (61.3)
Female	29 (38.7)
Age group of children	
8–11 Years	42 (56)
12–16 Years	33 (44)
Type Of cleft	
CL ± A	18 (24)
UCLP	33 (44)
BCLP	24 (32)
Occupation	
Private sector employee	52 (69.3)
Governmental employee	23 (30.6)
Type of parent	
Mother	48 (64)
Father	27 (36)

Table 2 shows the internal consistency of the overall scales for children and caregivers' (Cronbach's Alpha) was (0.70), and all other constructs ranged between (0.65 and 0.75).

**Table 2:** Internal consistency of the overall and subscales COHIP of the children with CL/P (n = 75) and caregivers (n = 75)

COHIP	Cronbach's Alpha	
	Children	Caregivers
Oral symptom (10 items)	0.75	0.73
Functional well-being (6 items)	0.74	0.71
Social well-being(8 items)	0.65	0.67
School environment (4items)	0.75	0.76
Self-image (6 items)	0.75	0.75
Overall COHIP (34 items)	0.70	0.70

COHIP scores for both children and caregivers' are shown in (Table 3). The mean overall score for children with CL/P was 89.41 ± 19.97 and for caregivers' was 94.34 ± 19.52. Caregivers' showed higher scores in all subscales than their children. There were significant difference between caregivers' and their children on the overall COHIP (*p* = 0.004) and oral symptoms subscale (*p* = 0.017).

**Table 3:** Overall and subscale COHIP scores and differences between children with CL/P (n = 75) and caregivers (n = 75)

COHIP scores	Mean (SD)		95% CI	t	*p* Value
	Children	Caregivers			
Oral symptoms	29.84 ± 6.11	31.22 ± 6.10	− 2.52, − 0.25	− 2.43	0.017*
Functional well-being	16.13 ± 4.19	16.32 ± 4.43	− 0.86, 0.49	− 0.55	0.583
Social well-being	17.18 ± 9.75	18.81 ± 8.77	− 3.5, 0.26	− 1.72	0.089
School environment	11.66 ± 3.92	12.34 ± 3.57	− 1.4, 0.04	− 1.88	0.063
Self-image	14.58 ± 4.33	15.64 ± 4.70	− 2.1,0.03	− 1.95	0.055
Overall COHIP	89.41 ± 19.97	94.34 ± 19.52	− 8.2, − 1.6	− 2.97	0.004*

*Statistically significant difference at p-value < 0.05

Intra-Class Correlation Coefficients as a measure of concordance between children with CL/P and their caregivers' reports are shown (Table 4). All correlations were statistically significant (*p* < 0.05).

**Table 4:** Correlations between subscales and overall scores of children with CL/P (n = 75) and caregivers (n = 75)

COHIP	Pearson	ICCs
Oral symptom	0.68**	0.81
Functional well-being	0.77**	0.87
Social Well-being	0.61**	0.76
School environment	0.66**	0.80
Self-image	0.47**	0.63
Overall COHIP	0.74**	0.85

**Correlation is significant with *p*-value < 0.01

ICCs indicates good to excellent agreement between child and caregivers', the correlation coefficient for the functional well-being was high, followed by oral symptoms and school-environment subscales. The correlation on self-image was relatively low.

Pearson correlation coefficients between subscale scores, overall, and general health in the parent and children groups are shown (Table 5).There were significant correlations between the subscales, overall, and general health. Similarly, the results of the children showed significant correlations between the subscales. School environment and social subscale proved to be significant predictor for general health only in the caregiver's sample.

**Table 5:** Correlations between overall, subscale and general health mean scores in caregivers (n = 75) (below the diagonal) and children (n = 75) (above the diagonal)

Caregivers	Children						
	Oral symptom	Functional well-being	Social well-being	School environment	Self-image	Overall COHIP	General health
Oral symptom	1	0.38**	0.22	0.20	0.19	0.57**	0.14
Functional well-being	0.46**	1	0.49**	0.34**	0.28*	0.69**	0.18
Social well-being	0.36**	0.53**	1	0.53**	0.44**	0.86**	0.39**
School environment	0.14	0.20	0.45**	1	0.33**	0.66**	0.36**
Self-image	0.29*	0.09	0.40**	0.42**	1	0.61**	0.54**
Overall COHIP	0.67**	0.67**	0.86**	0.57**	0.61**	1	0.46**
General health	0.17	0.06	0.23*	0.25*	0.59**	0.33**	1

**Correlation is significant at the 0.01, level (2-tailed)

*Correlation is significant at the 0.05 level (2-tailed)

Treatment expectations and the global health perceptions for both children and caregivers' (Table 6) showed that caregiver scored higher than their children, but the difference was statistically insignificant (*p* < 0.05)

**Table 6:** Results on treatment expectations and global health perception items children with CL/P (n = 75) and caregiver (n = 75)

COHIP (maximum possible scores	COHIP scores (mean ± SD)		t-test	*p *Value
	Children	Caregivers		
Treatment expectations	6.68 ± 1.42	6.94 ± 1.35	− 1.47	0.146
Global health perception	5.34 ± 1.53	5.50 ± 1.54	− 0.843	0.402

## Discussion

The objective of this study was to evaluate the OHRQoL of children with cleft lip and palate and their caregivers' using an Arabic version of the COHIP questionnaire. The internal consistency was acceptable in all subscales for children and caregivers', according to Streiner [23]. In the present study, OHRQoL perceptions were relatively high in both children and their caregivers'.

Caregivers' scored higher perception of OHRQoL than their children did in all the subscales. Statistically significant differences between children's and their caregivers' perceptions were found in oral symptoms subscale and the overall COHIP scores. These findings are consistent with a study conducted among the Dutch population [19] where significant differences were found between children and caregivers' in oral symptoms, emotional well-being, school-environment, and peer interaction subscale. Generally, caregivers' have low to a modest agreement with their children's QoL ratings [16, 24]. Konan et al. [25] reported a statistically significant difference between children and their caregivers' perceptions in self-image subscale. Wilson-Genderson [26] reported low agreement rates between child and caregiver when they asses the similarity in response between the children with craniofacial conditions and their caregivers'. Also Reissmann et al. found that parents' perception of their children OHRQoL is not accurate enough to detect problems in children age ranged between 7 and 17 years [27]. In contrast, other studies reported no differences between children and their caregivers' perceptions in any scale and the overall COHIP scores [20, 22, 28, 29].

Considering the importance of caregivers' perception to evaluate child's OHRQoL, the caregiver is responsible for educating the children about their health problem, and assess if the problem is affecting their life and needs attention [30]. From the results of the current study, the caregivers' perception of the child's OHRQoL differed from the child's perception. The caregivers' in the Sudanese sample reported higher scores than their children's suggesting that they believed that their children quality of life is better than what their children themselves reported. This indicates that children with orofacial clefts have opinions of their appearance, which provide an important aspect in their quality of life [22]. This raises an issue about the reliability of relying on caregivers' as a source of data regarding OHRQoL of the children with CL/P.

Previous studies also raised concerns about the interchangeability of caregiver and child reports regarding OHRQoL [28, 31]. Bos et al. [19] reported that caregivers' are limited in their interchangeability, a finding is in agreement with Wilson-Genderson [26] in which children had significantly higher scores than their caregivers'. Nolte et al. [32] and Jokovic et al. [11] support the recommendation that caregivers' reports should be used as complementary reports rather than the main data source.

Although the intraclass correlations were high but there were statistical significant differences in the overall and oral symptoms subscale which suggest those caregivers' reports could not replace those of the children themselves but can be considered as additional reports to those of their children. The high correlation value (0.86) between overall COHIP and social subscales suggesting the possible psychosocial problems that facing child with CLP as been reported previously by Alansari et al. [33] and Chimruang et al. [34] that such long treatment duration for children with CLP affects their psychological adjustment and self-satisfaction with their appearance.

In Sudan, there is no specialized center or cleft team to take care of children with cleft and their families. Most of the treatment being delivered by the different specialists at an individual level.As a result, many cleft children had been treated by surgical repair to the cleft defect without giving attention to other problems, which might have a negative impact on the OHRQoL in these children. Lack of education and information to the caregivers' may explain the difference in perception between them and their children.

The patients and caregivers' included in this study did not received any team care treatment. Most of these families are of low socioeconomic status. They find difficulties to accesses specialized health facilities in the treatment of CL/P and had to travel for long distances to receive services. Many of these families rely on treatments offered by charitable organizations and convoys that limit their services to surgical repair. This also contributes to low utilization. Similar concerns about the quality of life for children with cleft have been reported in other African countries [20, 35].

In this study, children with cleft rated their OHRQoL lower than their caregivers' rating. This is reflecting that children had concerns regarding their condition and the quality of treatment provided to them. This could be enhanced by educating families of children with cleft and the community about the importance of comprehensive multidisciplinary cleft care. It is also essential to raise community awareness regarding the cleft condition and the affected child. In addition, active engagement of the primary healthcare providers is needed to realize the need for team cleft care.

Since children with a CL/P may have a compromised aesthetic and functional well-being, they are at risk of a reduced OHRQoL [36]. This part of health care should be a focus area for more clinical researches to provide better treatment that improves their perception about the OHRQoL.

This study has several limitations. The number of children included in this study was small In addition, the sample included only one cleft care clinic therefore we cannot generalize these results to other cleft populations in Sudan. Another regarding the Arabic version of scale used is that the validation was limited to test–retest reliability and face validity.

We recommend that cleft care in Sudan should be revised to match the international protocols in regards to timing of treatment and multidisciplinary approach. A cleft registry should established to help in planning and provision of care for these children. Increasing the awareness level of the society on regard to children with CL/P, which will combat the possible stigma-related challenges and to educate parents in regards to cleft care and importance of seeking medical advice as early as time of childbirth. For future researches, a multi-center research involving clinics providing care to CL/P children.

## Conclusion

This study found that caregivers' had higher perceptions of oral symptoms and overall COHIP compared to their children themselves using an Arabic version of the COHIP that had acceptable internal consistency.

## Data Availability

The data that support the findings of this study are available from Ethical and Research Committee of Faculty of Dentistry, University of Science and Technology but restrictions apply to the availability of these data, which were used under license for the current study, and so are not publicly available. Data are however available from the author (MAA) upon request and with permission of Ethical and Research Committee of Faculty of Dentistry, University of Science and Technology.

## Abbreviations

WHO: The World Health Organization
OHRQoL: Oral health-related quality of life
OFCs: Orofacial clefts
CL/P: Cleft lip/palate
COHIP: The child oral health impact profile
QoL: Quality of life
ICCs: Intraclass correlation coefficients

## References

[1] McGrath C, Bedi R (1999). A review of the influences of oral health on the quality of life. Int J Health Promot Educ.

[2] World Organization of Health. Quality of Life Assessment. The WHOQOL Group, 1994. What Quality of Life? The WHOQOL Group. In: Health Promotion Glossary, p. 17. World Health Organization, Geneva, 1998. Available at: http://www.who.int/healthpromotion/about/HPR%20Glossary%201998.pdf. Accessed December 31, 2007

[3] Gift HC, Atchison KA, Dayton CM (1997). Conceptualizing oral health and oral health-related quality of life. Soc Sci Med.

[4] Topolski T, Edwards T, Patrick D (2005). how do adolescents with facial differences compare with other adolescents?. Cleft Palate Craniofac J.

[5] Suleiman AM, Hamzah ST, Abusalab MA, Samaan KT (2005). Prevalence of cleft lip and palate in a hospital-based population in the Sudan. Int J Pediatr Dent.

[6] Locker D, Jokovic A, Tompson B (2005). Health-related quality of life of children aged 11 to 14 years with orofacial conditions. Cleft Palate Craniofac J.

[7] Petersen PE. The World Oral Health Report 2003. Continuous improvement of oral health in the 21st century - the approach of the WHO Global Oral Health Programme. World Health Organization, 2003. Available at: http://www.who.int/oral_health/media/en/orh_report 03_en.pdf. Accessed December 31, 2007. 10.1046/j..2003.com122.x 15015736

[8] Wehby G, Ohsfeldt R, Murray J (2006). Health professionals' assessment of health-related quality of life values for oral clefting by age using a visual analogue scale method. Cleft Palate Craniofac J.

[9] Antonarakis GS, Patel RN, Tompson B (2013). Oral health-related quality of life in non-syndromic cleft lip and/or palate patients: a systematic review. Commun Dent Health.

[10] Jokovic A, Locker D, Stephens M, Kenny D, Tompson B, Guyatt G (2002). Validity and reliability of a questionnaire for measuring child oral-health-related quality of life. J Dent Res.

[11] Jokovic A, Locker D, Tompson B, Guyatt G (2004). Questionnaire for measuring oral health-related quality of life in eight- to ten-year-old children. Pediatr Dent.

[12] Slade GD, Spencer AJ (1994). Development and evaluation of the oral health impact profile. Commun Dent Health.

[13] Broder H, Wilson-Genderson M (2007). Reliability and convergent and discriminant validity of the child oral health impact profile (COHIP child's version). Commun Dent Oral Epidemiol.

[14] Broder H, Wilson-Genderson M, Sischo L (2012). Reliability and validity testing for the child oral health impact profile-reduced (COHIPSF 19). J Public Health Dent.

[15] Broder H, McGrath C, Cisneros G (2007). Questionnaire development: face validity and item impact testing of the child oral impact profile. Commun Dent Oral Epidemiol.

[16] Eiser C, Morse R (2001). A review of measures of quality of life for children with chronic illness. Arch Dis Child.

[17] Ennett ST, DeVellis BM, Earp JA, Kredich D, Warren RW, Wilhelm CL (1991). Disease experience and psychosocial adjustment in children with juvenile rheumatoid arthritis: children's versus mothers' reports. J Pediatr Psychol.

[18] Garber J, Van Slyke D, Walker L (1988). Concordance between mothers‟ and children‟s reports of somatic and emotional symptoms in patients with recurrent abdominal pain or emotional disorders. J Abnorm Child Psychol.

[19] Bos A, Prahl C (2011). Oral health-related quality of life in Dutch children with cleft lip and/or palate. Angle Orthod.

[20] Abebe ME, Deressa W, Oladugba V, Owais A, Hailu T, Abate F (2018). Oral health-related quality of life of children born with orofacial clefts in Ethiopia and their parents. Cleft Palate Craniofac J.

[21] Francois-Fiquet C, Dpouy M, Daoud S, Poli-Merol ML (2015). Cleft lip and palate: health-related quality of life French VSP-A scale for patients and their family about 51 families. Ann Chir Plast Esthet..

[22] Ward JA, Vig KW, Firestone AR, Mercado A, da Fonseca M, Johnston W (2013). Oral health-related quality of life in children with orofacial clefts. Cleft Palate Craniofac J.

[23] Streiner DL (2003). Starting at the beginning: an introduction to coefficient alpha and internal consistency. J Personal Assess.

[24] Achenbach T, McConaughy S, Howell C (1987). Child-adolescent behavioral and emotional problems: implications of cross-informant correlations for situational specificity. Psychol Bull.

[25] Konan P, Manosudprasit M, Pisek P, Pisek A, Wangsrimongkol T (2015). Oral health-related quality of life in children and young adolescent orthodontic cleft patients. J Med Assoc Thai.

[26] Wilson-Genderson M, Broder H, Phillips C (2007). Concordance between caregiver and child reports of child‟s oral health-related quality of life. Commun Dent Oral Epidemiol.

[27] Reissmann DR, John MT, Sagheri D, Siewald I (2017). Diagnostic accuracy of parents' ratings of thier childs' oral health -related quality of life. Qual life Res.

[28] Geels L, Kieffer J, Hoogstraten J, Prahl-Andersen B (2008). Oral health-related quality of life of children with craniofacial conditions. Cleft Palate Craniofac J.

[29] Munz SM, Edwards SP, Inglehart MR (2011). Oral health-related quality of life, and satisfaction with treatment and treatment outcomes of adolescents/young adults with cleft lip/palate: an exploration. Int J oral Maxillofacial Surg.

[30] Inglehart M, Bagramian R (2002). Oral health-related quality of life.

[31] Bos A, Hoogstraten J, Zentner A (2010). Perceptions of Dutch orthodontic patients and their parents on oral health-related quality of life. Angle Orthod.

[32] Nolte FM, Bos A, Prahl C (2019). Quality of life among dutch children with a cleft lip and/or cleft palate: a follow-up study. Cleft Palate Craniofac J.

[33] Alansari R, Bedos C, Allison P (2014). Living with cleft lip and palate: the treatment journey. Cleft Palate Craniofac J.

[34] Chimruang J, Soadmanee O, Srisilapanan P, Patjanasoontorn N, Nanthavanich N, Chuawanlee W (2011). A qualitative study of health-related quality of life and psychosocial adjustments of Thai adolescents with repaired cleft lips and palates. J Med Assoc Thai..

[35] Awoyale T, Onajole AT, Ogunnowo BE, Adeyemo WL, Wanyonyi KL, Butali A (2016). Quality of life of family caregivers of children with orofacial clefts in Nigeria: a mixed-method study. Oral Dis.

[36] Broder H, Wilson-Genderson M, Stein M, Crerand C, Rosenberg J, Riski J, et al. Child-caregiver concordance regarding children’s oral health-related quality of life. In: Paper presented at: Conference: IADR General Session. 2011.

